# Application of geostationary satellite and high-resolution meteorology data in estimating hourly PM_2.5_ levels during the Camp Fire episode in California

**DOI:** 10.1016/j.rse.2022.112890

**Published:** 2022-01-25

**Authors:** Bryan N. Vu, Jianzhao Bi, Wenhao Wang, Amy Huff, Shobha Kondragunta, Yang Liu

**Affiliations:** a Gangarosa Department of Environmental Health, Rollins School of Public Health, Emory University, Atlanta, GA, United States; b Department of Environmental & Occupational Health Sciences, School of Public Health, University of Washington, Seattle, WA, United States; c I.M. Systems Group, 5825 University Research Ct, Suite 3250, College Park, MD, United States; d Satellite Meteorology and Climatology Division, STAR Center for Satellite Applications and Research, National Oceanic and Atmospheric Administration, Washington, DC, United States; e Department of Environmental Health, Harvard T.H. Chan School of Public Health, Harvard University, Boston, MA, United States

**Keywords:** PM_2.5_, GOES16, Wildland fire, Remote sensing, AOD, SMOTE, Weighted Random Forest

## Abstract

Wildland fire smoke contains large amounts of PM_2.5_ that can traverse tens to hundreds of kilometers, resulting in significant deterioration of air quality and excess mortality and morbidity in downwind regions. Estimating PM_2.5_ levels while considering the impact of wildfire smoke has been challenging due to the lack of ground monitoring coverage near the smoke plumes. We aim to estimate total PM_2.5_ concentration during the Camp Fire episode, the deadliest wildland fire in California history. Our random forest (RF) model combines calibrated low-cost sensor data (PurpleAir) with regulatory monitor measurements (Air Quality System, AQS) to bolster ground observations, Geostationary Operational Environmental Satellite-16 (GOES-16)’s high temporal resolution to achieve hourly predictions, and oversampling techniques (Synthetic Minority Oversampling Technique, SMOTE) to reduce model underestimation at high PM_2.5_ levels. In addition, meteorological fields at 3 km resolution from the High-Resolution Rapid Refresh model and land use variables were also included in the model. Our AQS-only model achieved an out of bag (OOB) R^2^ (RMSE) of 0.84 (12.00 μg/m^3^) and spatial and temporal cross-validation (CV) R^2^ (RMSE) of 0.74 (16.28 μg/m^3^) and 0.73 (16.58 μg/m^3^), respectively. Our AQS + Weighted PurpleAir Model achieved OOB R^2^ (RMSE) of 0.86 (9.52 μg/m^3^) and spatial and temporal CV R^2^ (RMSE) of 0.75 (14.93 μg/m^3^) and 0.79 (11.89 μg/m^3^), respectively. Our AQS + Weighted PurpleAir + SMOTE Model achieved OOB R^2^ (RMSE) of 0.92 (10.44 μg/m^3^) and spatial and temporal CV R^2^ (RMSE) of 0.84 (12.36 μg/m^3^) and 0.85 (14.88 μg/m^3^), respectively. Hourly predictions from our model may aid in epidemiological investigations of intense and acute exposure to PM_2.5_ during the Camp Fire episode.

## 1. Introduction

Smoke from wildland fires can traverse tens to hundreds of kilometers away, carrying harmful pollutants that can affect adjacent and downwind communities resulting in excess morbidity and mortality. Although composition of wildland fire smoke is dependent on fuel type, temperature, and wind conditions, smoke from combustion of biomass is generally a mixture of particulate matter, carbon dioxide, water vapor, carbon monoxide, other organic chemicals, and trace minerals (Chow et al., 2006; Dominici et al., 2015; Sokolik et al., 2019). The size of the particulate matter from smoke emitted directly from wildland fires ranges considerably; however, larger particles are likely to deposit in the near field while smaller particles may remain in the atmosphere for days before depositing downwind. Particulate matter, especially PM_2.5_ (particulate mass of particles 2.5 μm or smaller in diameter), composes 90% of total particle mass emitted from wildland fires and has been linked to multiple adverse health outcomes including respiratory and cardiovascular diseases (Reid et al., 2019; Stone et al., 2019; Stowell et al., 2019a).

The health effects of PM_2.5_ have been widely documented in studies involving asthma, heart diseases, and premature death, and there is growing evidence that toxicity from particles generated by wildland fires differ from those emitted from other sources (Davila Cordova et al., 2020; Gan et al., 2020; Tapia et al., 2019; Tapia et al., 2020). For example, Leibel et al. found that the mean daily age-adjusted rate of respiratory emergency departments per 10,000 children in communities located downwind of a wildland fire increased from 55 in the week before the fire to 75 during the week of the fire (Leibel et al., 2020). Stowell et al. found that a 1 μg/m^3^ increase in wildland fire smoke PM_2.5_ was associated with an odds ratio of 1.08 in asthma emergency department visits yet found null associations with non-smoke PM_2.5_ (Stowell et al., 2019b). Furthermore, studies have also indicated that not only prolonged but also acute exposure to PM_2.5_ results in long lasting effects including persistent coughs, wheezing, and exacerbation of previous conditions such as asthma (Stone et al., 2019).

Human activities including energy production, industrial activities, and land-use change have led to increased greenhouse gas emissions and consequently climate change (IPCC, 2014). The Center for Research on the Epidemiology of Disasters reported 315 natural disasters globally in 2018 relating to climate change, with 10 of those cases being wildland fires (Fawzy et al., 2020). Climate change has also rendered California’s once temperate climate drought stricken while the dried forests act as the perfect fuel once a fire ignites (Goss et al., 2020; Williams et al., 2019). This problem is exacerbated by mismanagement of California’s forests and poor land use practices, resulting in overgrown and dried vegetation, acting as tinderboxes for wildland fires (LHC, 2018). The Camp Fire, which originated in Butte County in Northern California on November 8, 2018 and lasted for 17 days, for example, is considered the deadliest wildland fire in California history. It resulted in 85 casualties, 153,336 acres burnt, 18,804 structures destroyed, and completely destroyed two towns (U.S. Census Bureau, 2018). Economic losses from the Camp Fire stands at $16.65 billion, including $16.5 billion in insured losses and $150 million in firefighting costs (Reyes-Velarde, 2019). The Camp Fire not only resulted in massive economic losses, but may also result in increased long-term adverse health effects. Due to the intensity and duration of the fire, prolonged and cumulative exposure to fire smoke may result in progressive decline in lung function and increases the overall lifetime risk of heart disease and cancer (EPA, 2021). Additionally, California is marked by annual Santa Ana winds (SAWs) in the early fall, which is associated with the state’s most damaging wildland fires. One study suggested that under normal conditions, SAWs improve visibility inland by sweeping polluted air masses out to sea, resulting in lower PM_2.5_ levels near the coast (Aguilera et al., 2020). However, in the presence of fires upwind, SAWs have been shown to increase PM_2.5_ in areas downwind of the fires (Aguilera et al., 2020; Leibel et al., 2020). Due to the ability of fine particles in smoke to traverse great distances and the harmful effects previously documented in the literature, further research is needed to model the spatiotemporal trends of wildland fire PM_2.5_.

PM_2.5_ exposure assessments based solely on measurements from ground monitors such as the Environmental Protection Agency (EPA) Air Quality System (AQS) are often hindered by inadequate and uneven spatial coverage of the ground measurements. Recent studies also turned to real-time and near real-time PM_2.5_ sensors to improve the coverage of ground measurements (Mehadi et al., 2020). Studies estimating the PM_2.5_ concentrations during wildland fire episodes have relied on ground monitors, chemical transport models (CTMs), and more recently, remotely sensed data including satellite aerosol optical depth (AOD). AOD is a unitless measure of light extinction within the atmospheric column, and previous studies have shown its efficacy in predicting surface level PM_2.5_ (Bi et al., 2018; Hu et al., 2014; Liang et al., 2018; Meng et al., 2018; Vu et al., 2019). Various data fusion approaches have been proposed to model PM_2.5_ from wildland fire smoke (Baker et al., 2020; Guan et al., 2020; Mirzaei et al., 2020; Zou et al., 2019). For example, fusing ground measurements with satellite remote sensing data can significantly expand the scope of PM_2.5_ exposure modeling without substantially compromising the accuracy of the fused data, but is limited by the coverage of satellite data. Merging ground observations with CTM simulations tends to improve spatiotemporal coverage, and CTMs can also provide speciation information useful in targeting wildland fire smoke PM from other sources. However, the quality of the merged data in areas with limited numbers of monitors depends heavily on the accuracy of CTM simulations, which often contain large errors during fire events (Di et al., 2016; Diao et al., 2019; Koman et al., 2019).

In addition to estimating daily PM_2.5_ concentrations, the rapidly changing characteristics of fire smoke motivates the assessment of PM_2.5_ concentrations at the hourly level. For example, Marsha and Larkin relied on previous days’ satellite AOD and fire radiative power (FRP) to make hourly predictions at 10 km resolution. Their model achieved an R^2^ of 0.78 and a normalized RMSE of 4.9% (Marsha and Larkin, 2019). Sanchez-Balseca and Perez-Foguet used dynamic linear models that employ Gaussian Field principles to model hourly PM_2.5_ concentrations in wildland fire events (Sanchez-Balseca and Perez-Foguet, 2020). However, this approach has several limitations including the need for a sizable amount of ground monitors to both calibrate and validate the model, and the requirements of the presence of monitoring stations that acquire both PM_2.5_ and PM_10_ measurements to produce PM_2.5_/PM_10_ ratios needed in the modeling process. Li et al. used the Hybrid Single Particle Lagrangian Integrated Trajectory (HYSPLIT) model, coupled with emissions inventory and meteorological parameters to forecast hourly PM_2.5_ concentrations during the Camp Fire episode (Li et al., 2020b). The researchers configured the model with various combinations of biomass burning emissions data sets, plume rise schemes, meteorological inputs, mixing layer depth options, and vertical motion options to produce an ensemble that predicted PM_2.5_ at 0.1^◦^ resolution (Li et al., 2020b). However, the spatial resolution of this study is relatively coarse, and the ensemble model sometimes estimated PM_2.5_ levels 10 times higher than the EPA AQS measurements during the first 6 days of the fire and underestimated PM_2.5_ levels for the rest of the fire period, indicating large uncertainties. Such studies do not sufficiently account for the fine spatial and temporal resolution necessary to assess adverse health effects associated with wildland fire smoke.

With the advent of the Geostationary Operational Environmental Satellite-16 (GOES-16), satellite remotely sensed data including AOD, aerosol detection parameters, and fire spot characterization variables are now available at the sub-hour level to aid in modeling processes of events that will benefit from the aid of fine-scale temporal variables (Schmit et al., 2017). Data from GOES-16 have also been successfully utilized in estimating daily and hourly ambient PM_2.5_ (Zhang and Kondragunta, 2021). Recent studies have also shown the effectiveness of low-cost air quality sensors as promising supplements to regulatory ground monitors, by bolstering the number and coverage of ground observations during model training (Bi et al., 2020; Lin et al., 2020). For example, PurpleAir is a network of low-cost sensors providing continuous measurements of ambient PM_2.5_ and has been shown to accurately report air pollution measurements after calibration with gold-standard collocated monitors (Giordano et al., 2021; Johnson et al., 2020; Tryner et al., 2020). In this study, we reported a machine learning modeling method in conjunction with the Synthetic Minority Over-Sampling Technique (SMOTE) to fuse satellite remote sensing data, assimilated meteorological parameters, land use variables, and EPA AQS and PurpleAir measurements. The resulting model was used to estimate hourly PM_2.5_ levels at 3 × 5 km^2^ resolution during the Camp Fire period in California.

## 2. Materials and methods

### 2.1. Study domain

**Fig. 1.**
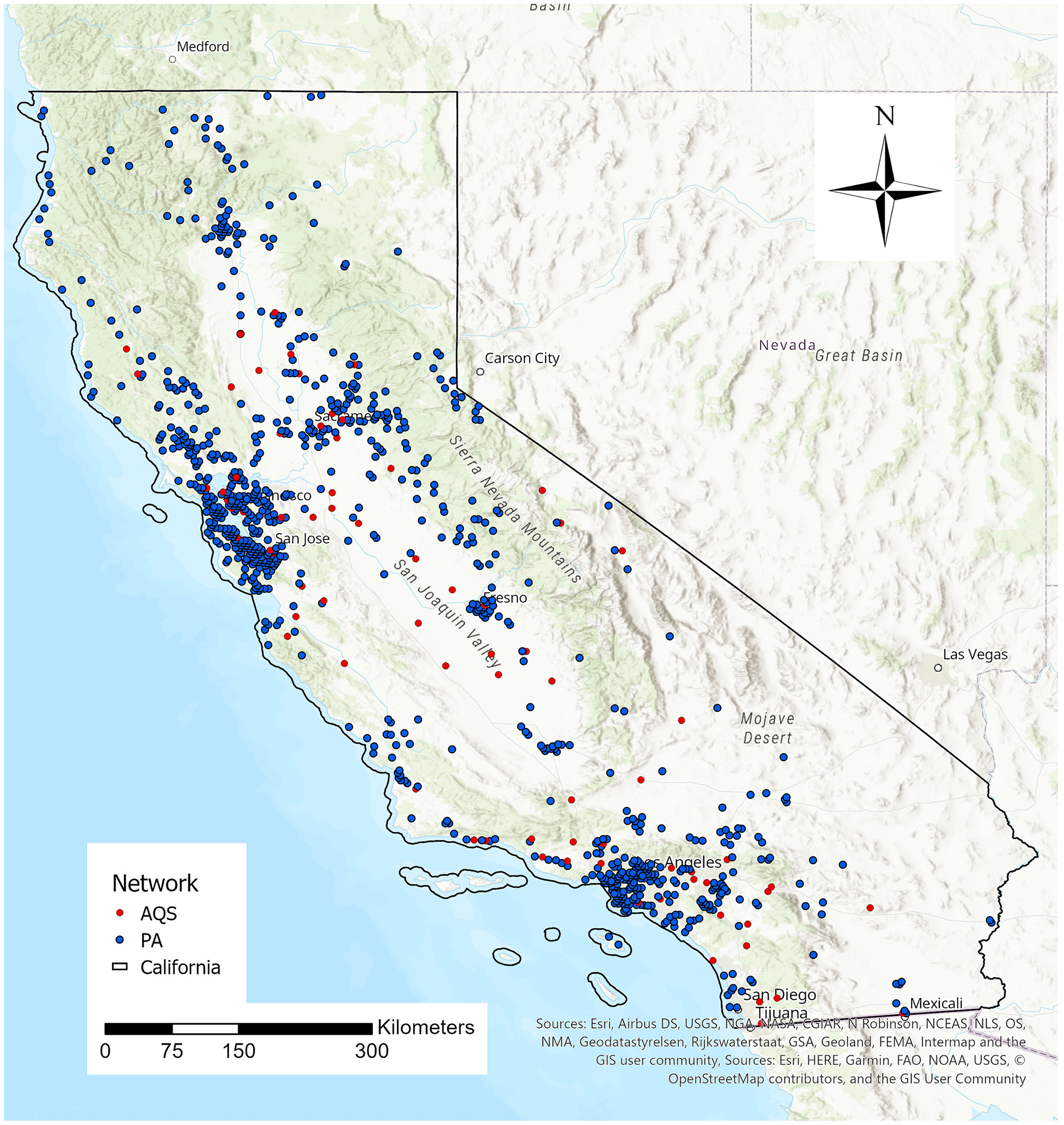
Study domain of California. EPA AQS monitors are pictured in red, PurpleAir sensors are in blue.

California is the third largest and the most populous state with 39 million residents spanning 423,970 km^2^ of the United States’ western region bordering the Pacific Ocean. The two most populous urban centers, the Greater Los Angeles Area in the south and the San Francisco Bay Area in the north, are the second and fifth largest metropolitan areas in the U.S., respectively. We created a modeling grid at 3 × 5 km^2^ spatial resolution for spatial alignment of all model parameters, and our study region includes 40,578 grid cells. Fig. 1 shows the study domain and location of ground monitors from the AQS and PurpleAir sensors.

### 2.2. Ground PM_2.5_ data

Hourly ground PM_2.5_ measurements were taken from the AQS network and the PurpleAir sensors from October 1 through November 30, 2018. AQS is a database of ground monitoring measurements maintained by the EPA (https://www.epa.gov/aqs) with 157 possible stations providing daily measurements in California, 108 of which provided 41,947 hourly measurements during the study period. PurpleAir is a citizen-based, real-time low-cost PM sensor network started in 2015 (https://www.purpleair.com/) with over 8000 sensors worldwide, measuring PM_1.0_, PM_2.5_ and PM_10_. Comparison of uncalibrated PurpleAir with AQS measurements found that on average, PurpleAir measurements were 1.9 μg/m^3^ higher than AQS measurements. Furthermore, the R^2^ and slope between the matched PurpleAir and AQS pairs ranged from 0.03 to 0.93 and 0.06 to 1.23, respectively, suggesting the need to calibrate the PurpleAir measurements before conducting any analyses (Bi et al., 2020). PurpleAir measurements in this study were calibrated through a method previously published, and subsequently 848 sensors in California contributed 207,103 hourly measurements during the study period (Bi et al., 2020). In short, calibration for the PurpleAir measurements was conducted through a geographically weighted regression (GWR). Temperature, relative humidity, operating time, uptime, and the PurpleAir measurement at each location was fitted against the AQS measurements based on colocation of each AQS/PurpleAir pair within a 500-m radius. Uptime (the time in which the sensor is in consecutive operation since the last boot time) and operating time (the duration between the time of the measurement and the installation of the sensor) is added to account for the durability of the sensor’s hardware which would correct for any time-changing sensor biases in downstream models. The calibration model had a 10-fold CV R^2^ of 0.78, and this process reduced PurpleAir’s systematic bias to ~0 μg/m^3^ and residual errors by 36%. Furthermore, each calibrated PurpleAir measurement was given a weight less than 1 in our model to control for their higher measurement error compared to AQS measurements which always receive a weight of 1. The overall weight of PurpleAir data was determined to be 0.15 and detailed analyses by Bi et al. showed that weighting the PurpleAir measurements can help reduce model underestimation at high PM_2.5_ levels. Because AQS measurements were considered gold standard in this study compared to PurpleAir, PurpleAir measurements were deleted in grid cells that contain both AQS and PurpleAir and only AQS measurements were kept. Furthermore, grid cells with more than one AQS or PurpleAir measurements were averaged to maintain one measurement per grid cell. On average, AQS monitors only provided six hourly measurements per station per day; therefore, the addition of PurpleAir measurements supplemented more ground measurements to ensure better model fitting results.

### 2.3. Satellite data

GOES-16 is a geostationary weather satellite operating in the east position at 75.2°W and provides high spatial and temporal resolution imagery through 16 spectral bands at visible and infrared wavelengths using Advanced Baseline Imager (ABI) (Nachamkin et al., 2009). Launched in November of 2016, GOES-16 was fully operational in December 2017 providing many different products at 2 km resolution near GOES-16’s final longitude, and up to 5 km in Western US. We collected AOD, aerosol detection binary variables, and fire spot detection variables during October and November of 2018. In our study period, fire spot detection and AOD products are available every 5 min and aerosol detection product is available every 15 min for the continental U.S. Aerosol detection product includes binary aerosol, dust, and smoke mask values. Fire (hot spot characterization) product provides four values: fire mask, a quantitative flag characterizing the quality of a particular pixel; temperature in kelvin; area in square kilometers; and radiative power in megawatts. All products were aggregated to the hourly level.

### 2.4. Meteorological variables

The High-Resolution Rapid Refresh (HRRR, https://rapidrefresh.noaa.gov/hrrr/) is a real-time atmospheric model run by the NOAA National Centers for Environmental Prediction that assimilates radar data every 15 min over a 1-h period to add further detail to the data provided by the hourly output from the Rapid Refresh model. HRRR has been shown to accurately simulate the observations of near-surface air and dew-point temperature (Lee et al., 2019). Furthermore, HRRR has been used to evaluate near-surface wind, temperature, and humidity conditions during wildland fire episodes, and when used in conjunction with GOES estimates, it proved beneficial in improving model performance in calculating geophysical processes such as actual evapotranspiration (Ha et al., 2020). In this study, we obtained hourly meteorological parameters including 2-m temperature, surface pressure, u- and v- wind, planetary boundary layer height, and relative humidity at 3-km spatial resolution from HRRR. HRRR data were joined to the GOES-16 grid through a nearest neighbor match while ensuring that no two HRRR data points were joined to the same GOES-16 grid cell.

### 2.5. Ancillary variables

Land-use parameters including percentage cultivated, barren, shrub, etc. were obtained from the 2011 National Land Cover Database at 30-m resolution (https://www.mrlc.gov/), elevation information was obtained from the Advanced Spaceborne Thermal Emission and Reflection Radiometer Global Digital Elevation (https://asterweb.jpl.nasa.gov/), and distances to nearest primary and secondary roads were computed from the U.S. Census TIGER/Line Shapefiles (https://www.census/). A convolutional layer was calculated for each ground PM_2.5_ measurement by taking an inverse-distance weighted average of the nearest five measurements from the same day and hour to ensure that the temporal window is fixed. We tested 5-, 10-, and 15-nearest neighbors; however, 10- and 15-nearest neighbors resulted in a convolutional layer that was closely correlated with the ground measurements. Regressions between the predictions from the model with these neighbors resulted in an R^2^ and slope above 0.97 and below 1.05 respectively, suggesting model overfitting. The convolutional layer was added to enhance spatial and temporal correlation between ground measurements. The distribution of AQS monitors is more heterogeneously spread across California compared to the distribution of PurpleAir sensors which are more clustered in densely populated regions such as the Bay Area and Los Angeles Area. Selection of too few nearby measurements may generate very similar synthetic measurements among a cluster of PurpleAir sensors, while selecting too many means that the synthetic estimate is generated from monitors that are too far apart and may carry more uncertainty. After a few rounds of testing, the convolutional layer was created using five nearest stations.

### 2.6. Modeling approach

A random forest (RF) model is a supervised machine learning ensemble method that aggregates sets of decision trees, or predictions, calculated from the best subset of predictors (Breiman, 2001). The RF model works by selecting a bootstrap sample from all observations with replacement, and subsequently selects the best set of predictors that provides the best split at each node. Advantages of the RF model include its accuracy in learning and classifying features, ability to include large numbers of predictors, and ability to provide variable importance measures that explain the relative contribution of each predictor. Furthermore, individual weights may be assigned to each observation in instances when certain observations are favored over others (e.g., higher accuracy). The RF model has two major hyper-parameters to tune, number of decision trees to grow (*n*_*tree*_), and the number of predictors randomly tried at each split (*m*_*try*_), as well as the depth and complexity of the trees.

We trained the RF model through three different approaches. In the first approach, a RF model is trained with AQS-only measurements. The second approach incorporated PurpleAir measurements to bolster the number of ground observations and enhance measurements of high PM_2.5_ concentrations near the Camp Fire site. In this RF model, full weight is given to AQS measurements and 15% weight is given to PurpleAir measurements. The lower weight of the PurpleAir measurements reflects the higher measurement errors of PurpleAir sensors as well as the lack of consideration in spatial representativeness of this citizen-based network. A more detailed discussion on how the PurpleAir measurements were calibrated and measurement errors is provided elsewhere (Bi et al., 2020). Because high PM_2.5_ concentrations account for only a small fraction of the AQS and PurpleAir data, the third approach applies a Synthetic Minority Over-sampling Technique (SMOTE) to the model training dataset to enhance model performance at high PM_2.5_ levels. SMOTE is a statistical technique that generates synthetic samples using information about the minority class available in the training data (Blagus and Lusa, 2013). In this study, we set the minority class as any ground measurement at or above 100 μg/m^3^ (2% of the total number of ground observations), nearly three times the daily U.S. national ambient air quality standard (NAAQS) of 35 μg/m^3^. For each measurement in the minority class, the SMOTE function synthetically produces an observation along with its predictors from the five nearest neighbors (Blagus and Lusa, 2013). Due to the small number of minority observations in our model training dataset, the application of SMOTE enhances the distribution of ground measurements and does not skew the distribution in any way. All three model approaches included the same predictors shown in Table S1 and the same *m*_*try*_ (set as default, the square root of the number of predictors rounded up, 6) and *ntree* (500), a minimum node size of 5, and a max depth of 20. Finally, a 10-fold temporal cross-validation (CV) technique and a leave-ten-out spatial CV are implemented in all three approaches to evaluate model performance. The 10-fold temporal CV works by sorting the model fitting dataset by date and time and dividing the total number of observations into 10 segments. Measurements from nine segments are used to train the model and the remaining segment is used to test predictions. This process is repeated 10 times to achieve predictions for all measurements (Murray et al., 2019; Young et al., 2016). For the leave-ten-out spatial CV, the model fitting dataset is divided into 96 groups of 10 monitors ensuring that each group of 10 monitors are spatially heterogeneous. Ninety-five groups were used to train the model while the remaining 10 monitors are used to to test predictions. This process is repeated until all measurements have a prediction. For the third model, we applied the SMOTE technique after dividing the data into segments to ensure that information from the training dataset is not influenced by information from the validation dataset. Model performance is based on the out of bag (OOB) R^2^ and CV R^2^. OOB R^2^ is calculated from the samples left out of the bootstrapped sample in the training dataset and is computed as the number of correctly predicted rows from the out of bag sample. In contrast, the CV R^2^ is calculated from a portion of the original training dataset that is intentionally set aside for predictions and validation. All data analyses were conducted in R Studio version 3.6.2 and mapping was conducted in ArcGIS version 10.7.1.

## 3. Results

**Fig. 2.**
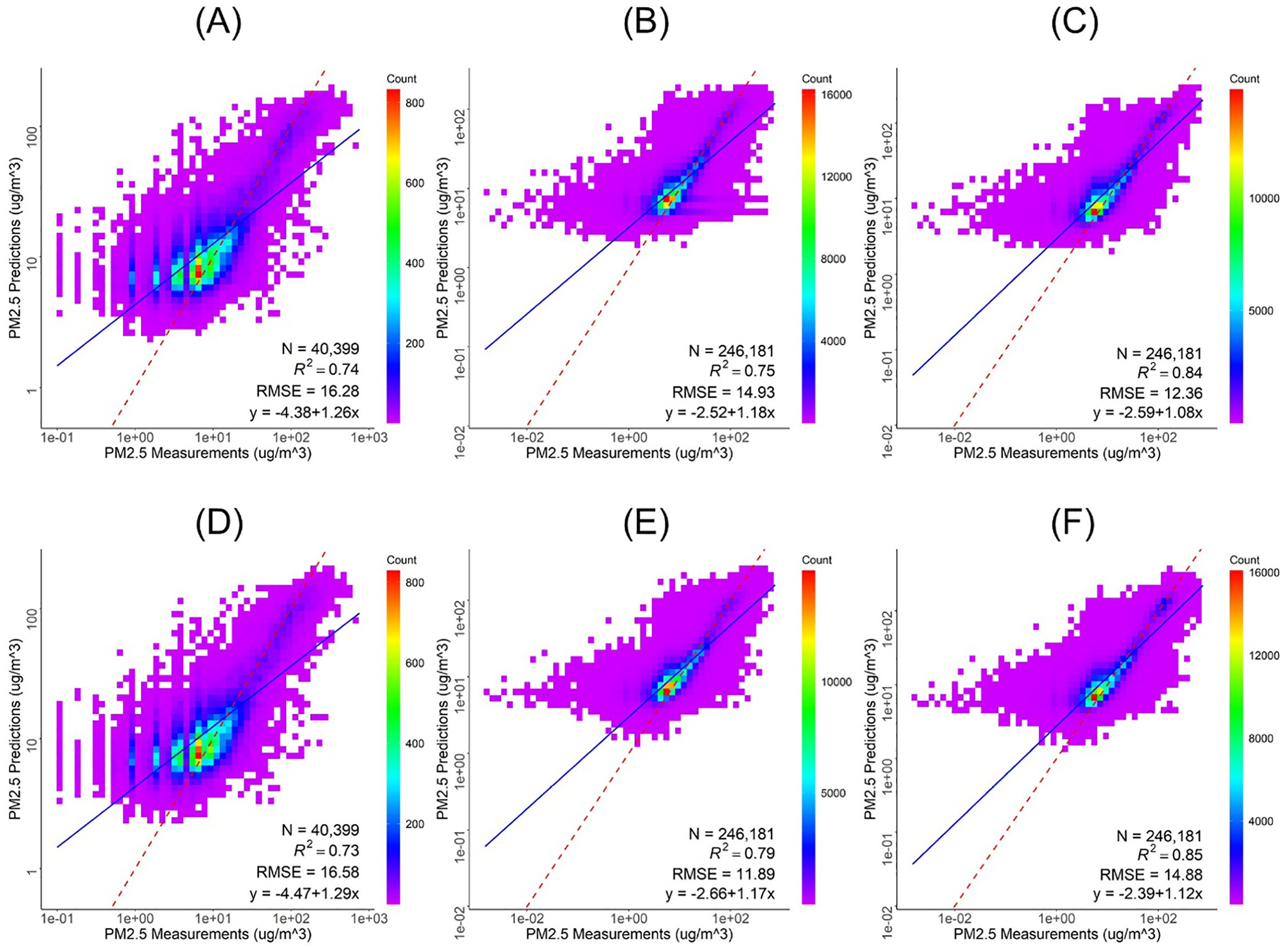
Panel of density scatter plots of 10-fold spatial CV measured vs. predicted PM 2.5 concentrations from (A) AQS-only Model, (B) AQS + Weighted PurpleAir Model, and (C) AQS + Weighted PurpleAir + SMOTE Model, and the 10-fold temporal cross-validation measured vs. predicted PM_2.5_ concentrations from the three models (D) AQS-only Model, (E) AQS + Weighted PurpleAir Model, and (F) AQS + Weighted PurpleAir + SMOTE Model. The dotted red line designates the slope and intercept while the solid blue line designates a 0 intercept with a slope of 1.

Panels A, B, and C in Fig. 2 show the scatter plots of measured vs. model predicted PM_2.5_ concentrations for the leave-ten-out cross-validation from the three models, A) AQS-only Model, B) AQS + Weighted PurpleAir Model, and C) AQS + Weighted PurpleAir Model + SMOTE Model. Panels D, E, and F in Fig. 2 show the scatter plots of the 10-fold temporal cross-validation from the three models.

### 3.1. AQS-only model

In total, there were 40,399 grid-averaged hourly AQS observations spanning the modeling period, between October 1 and November 30, 2018 with PM_2.5_ ranging from 0.1 to 657 μg/m^3^. The model out of bag (OOB) R^2^ is 0.84 (RMSE = 12.00 μg/m^3^), and the spatial CV R^2^ (RMSE) is 0.74 (16.28 μg/m^3^) and the 10-fold temporal CV R^2^ (RMSE) is 0.73 (16.58 μg/m^3^). Variable importance ranking from this RF model indicates that aside from the convolutional layer, elevation, pressure, and percent herbaceous land cover were the top three predictors. GOES-16 AOD is the 12^th^ most important predictor while detection of aerosol, detection of smoke, and smoke mask ranked 21^st^, 22^nd^, and 27^th^, respectively. HRRR variables including pressure and planetary boundary layer height (PBLH) rank 1^st^ and 8^th^, respectively. Other HRRR variables such as friction velocity, wind components, and radiation flux vary after rank 10.

### 3.2. AQS + Weighted PurpleAir Model

In total, there were 246,181 grid-averaged hourly combined AQS and PurpleAir observations during the study period with ground level PM_2.5_ measurements ranging from 0 to 707 μg/m^3^. The model OOB R^2^ is 0.86 (RMSE = 9.52 μg/m^3^) showing some improvement when PurpleAir measurements are included both in terms of model fit and residual error, likely since PurpleAir measurements captured more higher values. The spatial CV resulted in an R^2^ (RMSE) of 0.75 (14.93 μg/m^3^) and 10-fold temporal CV resulted in an R^2^ (RMSE) of 0.79 (11.89 μg/m^3^). Variable importance ranking from this model is similar to the AQS-only Model, with the convolutional layer, pressure, nearest distance to roads and elevation being the top four predictors. GOES-16 AOD ranked 14^th^ highest in importance while detection of aerosol, smoke, and smoke mask ranked 22^nd^, 26^th^, and 29^th^, respectively. Similar to the AQS-only Model, pressure and PBLH rank 1^st^ and 8^th^, respectively.

### 3.3. AQS + Weighted PurpleAir + SMOTE Model

Of the 246,181 hourly combined AQS and PurpleAir PM_2.5_ observations, 4819 are at or above the 100 μg/m^3^ minority cutoff. The SMOTE application produced an additional two synthetic observation for each minority observation, resulting in 255,819 total grid-averaged hourly combined AQS and PurpleAir observations. The OOB R^2^ is 0.92 (RMSE = 10.44 μg/m^3^), and spatial and temporal CV R^2^ (RMSE) were 0.84 (12.36 μg/m^3^) and 0.85 (14.88 μg/m^3^), respectively, based on the original 246,181 observations. These results indicate a sizeable decrease in the residual errors compared to the AQS-only Model due to the application of SMOTE. Variable importance shows that aside from the convolutional layer, nearest distance to roads, pressure, and 10-m u-wind component were the most important predictors in this model. GOES-16 AOD ranked 9^th^ highest important while smoke mask, detection of smoke and detection of aerosol ranked 14^th^, 25^th^, and 26^th^, respectively. In this model, HRRR pressure ranked 2^nd^, 10-m U-wind component and PBHL ranked 4^th^ and 5^th^, respectively, while 10-m V-wind component ranked 10^th^. In all three models, the binary dust detection, area, temperature, and radiative power of fire spot variables consistently ranked lowest in the models and were consequently excluded from all models. Table S2 of the supplemental shows the variable importance ranking for all three models.

**Fig. 3.**
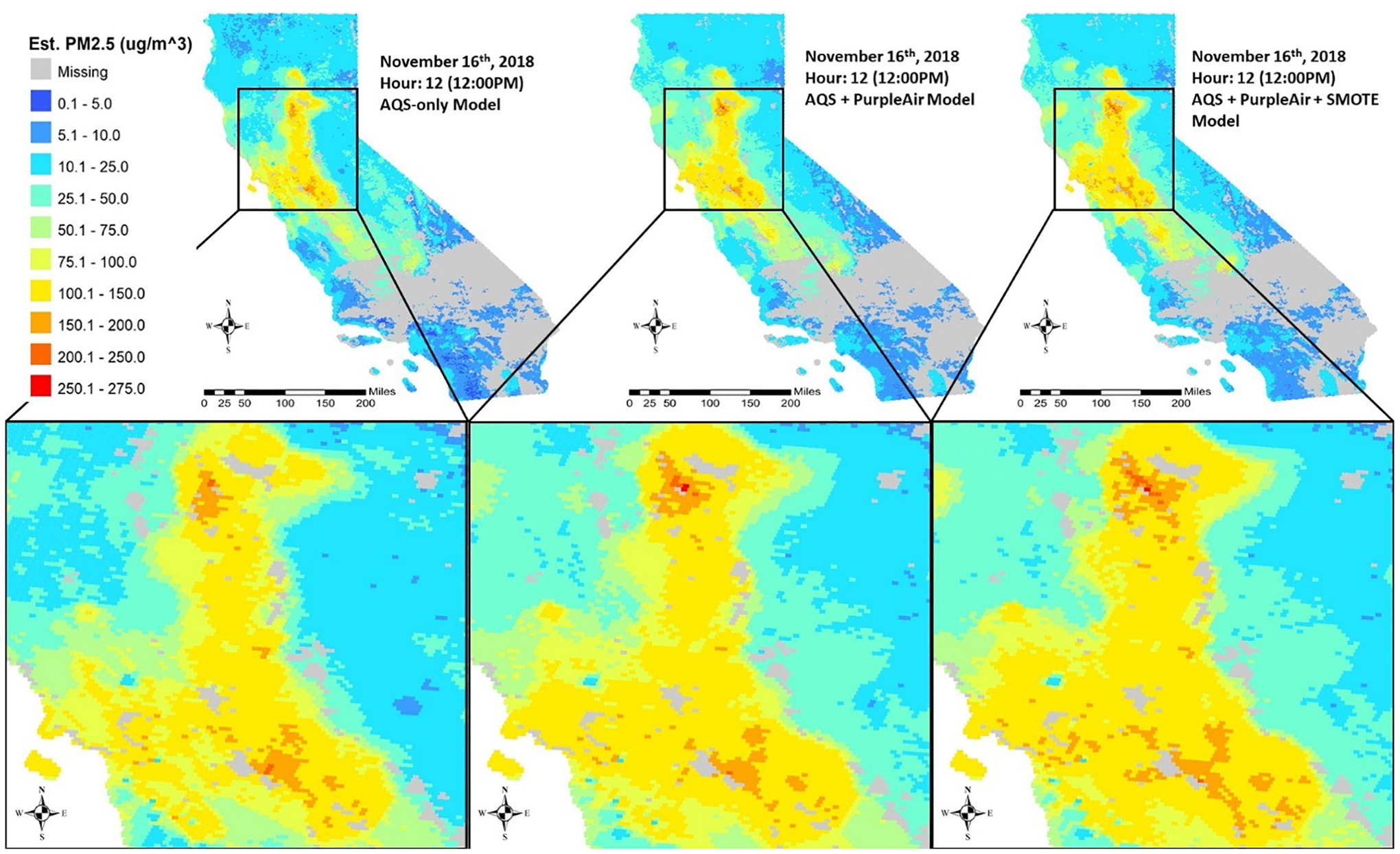
Predictions from all three models (AQS-only, AQS + Weighted PurpleAir, AQS + Weighted PurpleAir + SMOTE) at 12:00 pm on November 16th, 2018. Area of the smoke plume remains the same in all models; however, PM 2.5 levels increase as PurpleAir and SMOTE is added.

As a sensitivity analysis, predictions from all three models were made for the same hour on the day the Camp Fire reached its peak, November 16th, to assess model performance and prediction capabilities. Fig. 3 shows the estimated PM_2.5_ at noon on November 16th. Although the shape of the smoke plume does not change, PM_2.5_ estimates increased with the addition of PurpleAir and SMOTE.

**Fig. 4.**
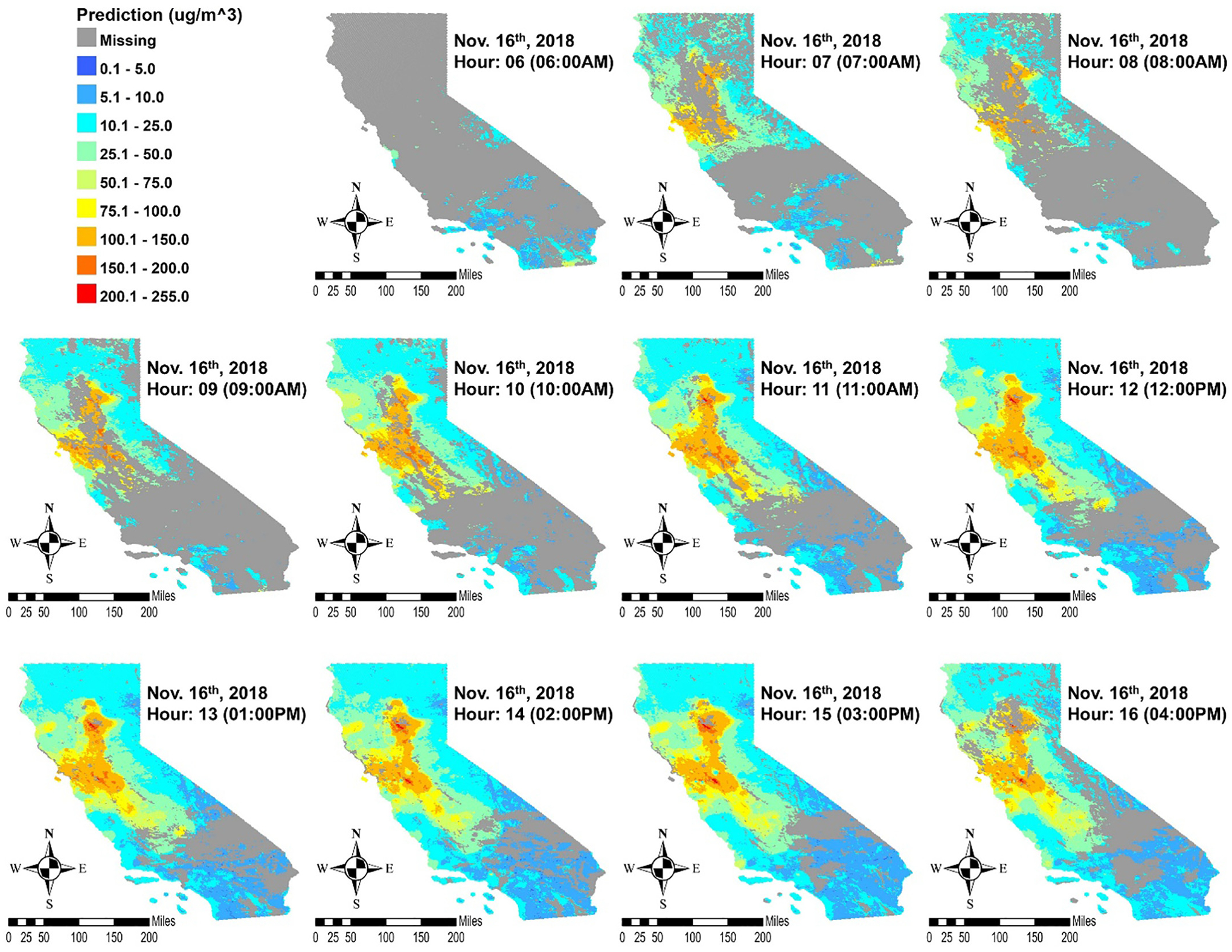
Hourly prediction maps of PM 2.5 in μg/m^3^ from the weighted RF and SMOTE model in California on November 16, 2018. Recorded ground measurements were highest on this day.

**Fig. 5.**
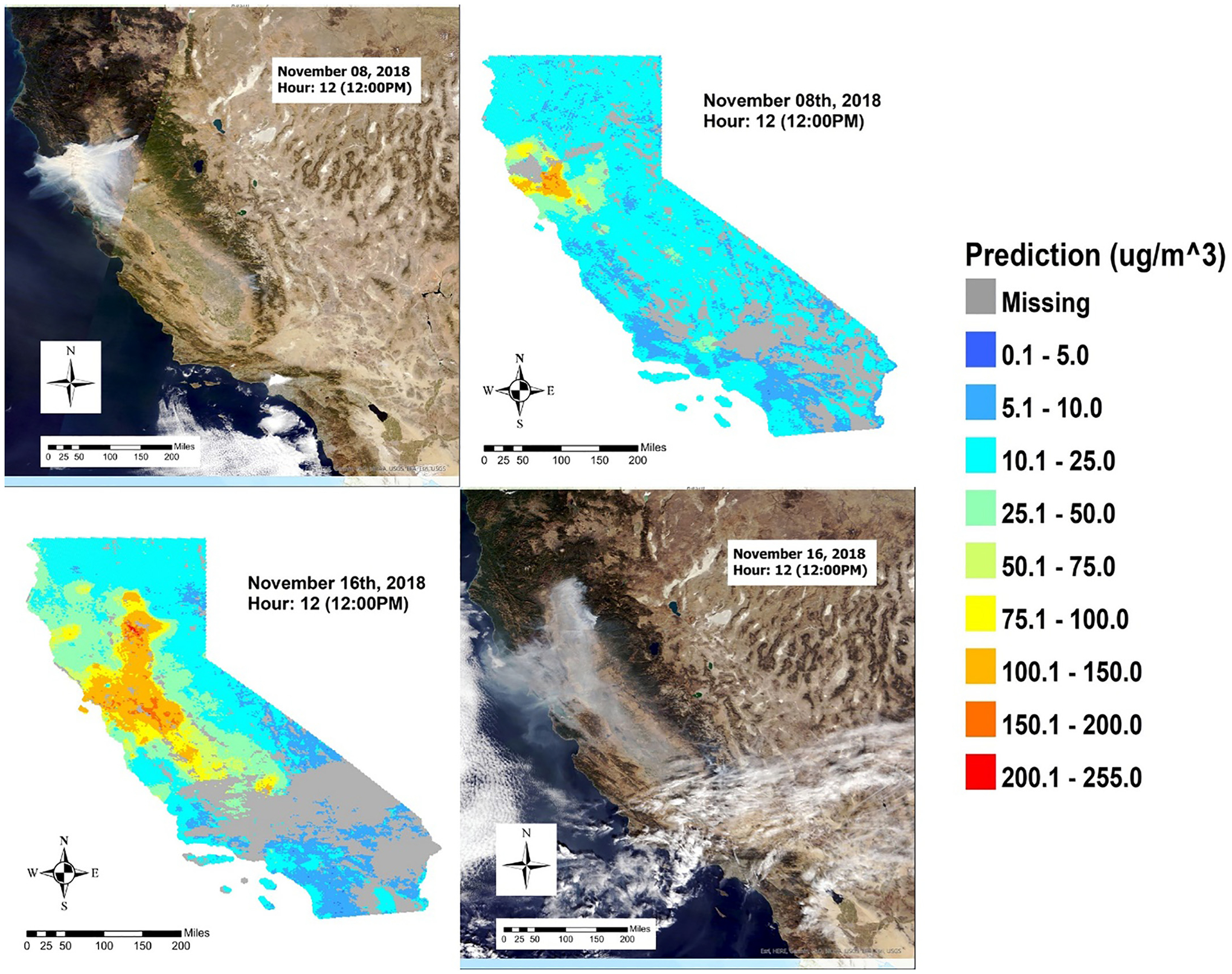
Comparison of hourly prediction maps of PM 2.5 in μg/m^3^ with the true color composite images from MODIS at noontime on November 8 and November 16, the day the Camp Fire started and the day with the highest recorded ground measurements, respectively.

Hourly predictions were made for the extent of California in grid cells where and when all predictors are present. Fig. 4 shows an example of hourly PM_2.5_ predictions by the weighted RF and SMOTE model on November 16, the day ground measurements recorded the highest levels of PM_2.5_ from 6 am to 4 pm PST. Furthermore, comparison of hourly prediction maps with the true color composite images from MODIS, suggests predictions from the weighted RF and SMOTE model largely aligns with the true-color images of the smoke plumes. Fig. 5 shows this comparison with images at noontime on November 8 and November 16. Minor differences in the true-color images and our prediction maps could be caused by our model capturing the PM_2.5_ levels on the surface rather than the column-integrated smoke plumes captured in the satellite image.

## 4. Discussion

In this study, we developed a model to predict surface wildland fire PM_2.5_ concentrations during the Camp Fire using three approaches. To the best of our knowledge, this is the first study that integrated low-cost sensor data to bolster ground observations and GOES-16 satellite remote sensing data to achieve high spatiotemporal resolution concurrently. To date, many studies documenting the effects of wildland fires on human health have focused on exposures ranging from days to months, often limited by the lack of fine-temporal exposure estimates since most satellite-based machine learning models published in the literature estimated daily, monthly, or annual mean PM_2.5_ levels in ambient conditions over a relatively long study period (Agyapong et al., 2020; Liang et al., 2018; Liu et al., 2009; Ontawong et al., 2020; Vu et al., 2019; Woo et al., 2020). Especially in California, where wildland fires seem to ravage both the northern and southern regions repeatedly each year with growing intensity as climate change progresses, evidence-based guidelines regarding vulnerable populations are needed to mitigate risks and parse out the adverse health effects of wildland fire smoke from those caused by other environmental hazards (Maxmen, 2019).

Results from the AQS-only Model show that using EPA’s AQS ground measurements alone to model wildland fire PM_2.5_ may underestimate PM_2.5_ levels. Model performance is adversely affected by lack of extensive hourly measurements as well as the lack of AQS monitors near the Camp Fire site to pick up high concentrations. Although AQS monitors are relatively evenly distributed across California, there are only a few located near the site of the Camp Fire. Integrating PurpleAir measurements increased ground observations by over 500%, bolstered the number of measurements at and around the Camp Fire, and improved both model fitting OOB R^2^ and RMSE. Even though the model fitting OOB R^2^ only improved by 2%, the intercept reduced by 23% from 1.62 to 1.25. Furthermore, the average PM_2.5_ prediction based on the AQS-only Model on November 16th at noon was 30.6 μg/m^3^ while the average PM_2.5_ prediction from the AQS + PurpleAir Model on the same day and time was 34.3 μg/m^3^ suggesting that the addition of PurpleAir measurements increased the predictions from the AQS-only model. However, these improvements are only minimal since there are still uncertainties in the low-cost sensor measurements due to the light-scattering principle associated with laser particle counters and manufacturing calibration and maintenance. For example, uncertainty may be present in the monitors recording incorrect particle counts and in the conversion between particle counts and mass concentrations. Furthermore, sensors may degrade over time. As a result, data quality may differ based on sensor location and condition. Nonetheless, calibration by Bi et al. indicates that the density of the PurpleAir network partially offsets the impact of measurement errors. As a result, a weight of 15% is given to the PurpleAir measurements, allowing AQS measurements to still have a major role in model training. Predictions in Fig. 3 show that the addition of PurpleAir intensified the PM_2.5_ estimates in the north where the fire originated and also in the west due to Santa Ana winds blowing the smoke from east to west. Although the addition of PurpleAir measurements augmented the number of high values, the model still underestimates at high PM_2.5_ levels. Nonetheless, the addition of PurpleAir measurements enables us to calibrate the model for more accurate predictions throughout the entire study domain, especially where the fire originated and downwind from it.

Due to the nature of modeling a wildland fire event in a large domain, the distribution of the ground measurements is right-skewed since the majority of monitors and subsequently their measurements are outside the vicinity of the fire smoke. To compensate, we applied the SMOTE technique to artificially inflate the high values and improve model performance. The duplication of measurements at or above 100 μg/m^3^ ensures that these measurements are more wildland fire-related. Additionally, SMOTE synthetically duplicates the high observations by inverse weighting the nearest five neighbors which also ensure that these duplicates have similar predictors without being exactly identical. The number of high measurements after duplication remains well below 6% of the total number of observations, which we deem as not significantly altering the original distribution. There were 3.7 times more PurpleAir measurements at or above the minority cut off compared to AQS and the implementation of SMOTE improves model performance even though PurpleAir measurements were still given only 15% weight compared to AQS. We utilized a 10-fold leave-10-monitors out spatial CV and a temporal CV to gauge model performance. Monitors neighboring the wildland fire will inevitably measure higher PM_2.5_ levels compared to monitors across the study domain, and these monitors will also measure high values consistently through the extent of the wildland fire period. Consequently, the spatial and temporal CV will help determine if model performance is influenced by information from both nearby hours and nearby monitors. Although CV results suggest that all three models underestimate the high levels of the wildland fire PM_2.5_, predictions from each model indicate the need to incorporate both PurpleAir measurements and the SMOTE technique. Our full model captures both the spatial extent and the intensity of the smoke plume produced by the wildfire that the AQS-only Model inadequately achieved.

In the end, the model that utilized both AQS and PurpleAir measurements with the incorporation of a weighted sampling scheme and SMOTE is our best performing model. By utilizing low-cost sensor measurements from PurpleAir, we are able to expand both the spatial and temporal coverage of the ground measurements to improve model calibration and performance in predicting wildland fire PM_2.5_. Using a machine learning model with a weighted sampling scheme ensures that the gold-standard bearer AQS measurements are still prominently featured in the models and the implementation of SMOTE allowed us to slightly inflate high levels to reduce model underestimation. To date, few studies exist that predict wildland fire PM_2.5_ due to lack of ground measurements and good predictor variables. A recent model by Li et al. utilized deep learning techniques to predict weekly PM_2.5_ during a 10-year timespan between 2008 and 2017 in California using MAIAC AOD, variables from MERRA-2, and meteorological and land cover parameters (Li et al., 2020a). Although their model did not focus specifically on wildland fire PM_2.5_, it achieved a similar training R^2^ of 0.94 and validation R^2^ of 0.82 (Li et al., 2020a), indicating that our model is able to perform sufficiently compared to those with similar predictors in the same region. Furthermore, our model is capable of estimating hourly PM_2.5_ levels during the Camp Fire episode. Therefore, results from our model will enable researchers to investigate the spatial extent at which wildland fire smoke PM_2.5_ traverses as well as the acute temporal fluctuations in concentrations and link this exposure to adverse health outcomes targeted at communities downwind of the event.

The models presented in this study aim to estimate PM_2.5_ during a short but intense wildland fire event where the PM_2.5_ emission source profile may be much more complex than those reported in previous research. Fig. 5 shows that the spatial pattern of fire smoke dominated most of the study domain on November 16th; however, the pattern is limited to only the origin of the fire on November 8th when the fire started. Thus, the smoke plume dominates the distribution of high PM_2.5_ concentrations over a limited geographical region while the rest of California was still primarily affected by ambient sources such as land cover types, elevation, distance to road, and population density which are higher ranked in model importance output. Furthermore, since the models were developed at the hourly level, sudden meteorological changes such as wind speed/direction and boundary layer height may have major impact on the spatial pattern of PM_2.5_, which may explain the fluctuation in importance for these variables.

Comparison of our hourly predictions to true color composite images from MODIS Aqua show very similar smoke plumes in spatial extent, indicating good model performance. Although predictions are limited to sunlight hours due to the availability of AOD and other GOES-16 predictors used in this study, our model is able to pick up the heterogeneity in PM_2.5_ distribution even inside the smoke plume. However, there are a few limitations. First, GOES-16 is the east position geostationary satellite with a skewed view of the Pacific West. As a result, many of the quality flags associated with some of the variables such as AOD suggests low quality. A possible reason for the low quality AOD is the geometrics of the geostationary satellite (Zhang et al., 2020). Unfortunately, GOES-17, the west position geostationary satellite was not suitable for scientific analyses until January of 2019, after the Camp Fire event. Furthermore, there is also uncertainty if GOES-16 is able to accurately pick up AOD directly inside the smoke plume due to the heavy aerosol loading. Therefore, we are unsure if the missingness is directly due to failure to retrieve an AOD value or if the true AOD value is higher than GOES-16’s capable range. Future research may consider using GOES-16’s visible band reflectance as input instead of AOD. Although the geostationary satellite’s variables including AOD are not highly ranked in the models and the overall model performance metrics such as the R^2^ did not significantly changed, these variables are able to pick up spatial variability in air pollution especially around hotspots that the meteorological variables may not be able to capture. Furthermore, even though the meteorological variables are ranked higher in our models, the spatial contrast of these variables may have been lost due to data processes including interpolation which creates smoother data surfaces. Without the inclusion of the geostationary satellite’s data, the prediction surfaces would only vary temporally from hour to hour and not spatially from place to place (Liu et al., 2009). Second, although the integration of SMOTE within the model improves the OOB R^2^; the implementation of such an approach is arbitrary. Other methods to deal with imbalanced data include under-sampling; however, removing instances in the majority class when two or more observations are similar may result in loss of information.

## 5. Conclusion

The present study is the first to incorporate high temporal resolution geostationary satellite data with low-cost sensors to model wildland fire PM_2.5_ during a major wildland fire event, the Camp Fire episode in California. We found that using only ground observations from EPA’s AQS network alone was not sufficient for modeling hourly PM_2.5_, and that the addition of PurpleAir low-cost sensors not only bolstered our number of observations but also improved the R^2^ and RMSE. Furthermore, the implementation of SMOTE to synthetically enhance high values in our model training dataset further enhanced the model’s ability to estimate high PM_2.5_ values. Predictions from our model may be used for epidemiological studies investigating both long-term cumulative exposure to wildland fire PM_2.5_ and also acute intense short-term exposure as well.

## Supplementary Material

supplement
