# Supplementary material for: Application of geostationary satellite and high-resolution meteorology data in estimating hourly PM2.5 levels during the Camp Fire episode in California
Source: Remote Sens Environ. Author manuscript; Available in PMC 2023 Apr 7. (PMC10081518; doi:10.1016/j.rse.2022.112890)
Supplement: supplement [file NIHMS1879134-supplement-supplement.docx]

**Supplemental Materials**

All three model approaches utilize the same variables listed below in Table S1. As well as the same parameter specifications in the random forest model, *m_try_* set to default (square root of the total number of parameters rounded up, 6) and *n_tree_* of 500.

Table S1. Predictor variables used in random forest models of all three approaches.

| **GOES-16** | **HRRR** | **Land-use** |
| --- | --- | --- |
| AOD | Planetary boundary layer height (PBLH) | Elevation |
| Aerosol detection | Pressure | Nearest distance to roads |
| Smoke detection | Upward longwave radiation flux (ULWRF) | Population |
| Smoke mask flag | Downward longwave radiation flux (DLWRF) | % of shrub lands |
|  | Sensible heat net flux (SHNF) | % of herbaceous areas |
| **Ancillary** | Upward shortwave radiation flux (USWRF) | % of developed areas |
| Convolutional layer | Downward shortwave radiation flux (DSWRF) | % of cultivated areas |
|  | 10-meter U-wind component | % of forest |
|  | 10-meter V-wind component |  |
|  | Visibility |  |
|  | Total cloud cover (TCC) |  |
|  | Low cloud cover (LCC) |  |
|  | Specific humidity |  |
|  | Relative humidity |  |
|  | Friction velocity |  |
|  | Dew point |  |
|  | 2-meter temperature |  |
|  | Wind speed |  |
|  | Wind direction |  |
|  | Wind gust |  |

One of the major advantages of the random forest (RF) model is the output of variable importance from the model, which indicates how much each variable in the model affects the root mean square error (RMSE). Table S2 lists the variable importance output for each of the three models.

Table S2. Variable importance output from the three Random Forest Models.

| **Rank** | **AQS-only Model** | **AQS+PurpleAir and Weighted Model** | **AQS+PurpleAir and Weighted + SMOTE Model** |
| --- | --- | --- | --- |
| 1 | Convolutional Layer | Convolutional Layer | Convolutional Layer |
| 2 | Pressure | Pressure | Road Distance |
| 3 | Elevation | Road Distance | Pressure |
| 4 | % of herbaceous | Elevation | % herbaceous |
| 5 | Population | % herbaceous | U-wind |
| 6 | Road Distance | % shrub | PBLH |
| 7 | % of shrub | % developed | Elevation |
| 8 | % of developed | % forest | % shrub |
| 9 | PBHL | PBLH | SHNF |
| 10 | % barren | Population | AOD |
| 11 | % water | U-wind | V-wind |
| 12 | V-wind | % water | % barren |
| 13 | AOD | % barren | % water |
| 14 | % cultivated | % cultivated | ULWRF |
| 15 | Temperature | AOD | Det. Mask |
| 16 | % forest | DLWRF | Rel. Humidity |
| 17 | DSWRF | Specific Humidity | % cultivated |
| 18 | ULWRF | Temperature | Friction Velocity |
| 19 | SWRF | ULWRF | DSWRF |
| 20 | Specific Humidity | V-wind | TCC |
| 21 | Dew Point | TCC | DLWRF |
| 22 | Det. AOD | Wind Direction | Specific Humidity |
| 23 | Det. Smoke | Det. AOD | Visibility |
| 24 | Friction Velocity | USWRF | Dew Point |
| 25 | SHNF | Visibility | Temperature |
| 26 | DLWRF | SHNF | Det. Smoke |
| 27 | Rel. Humidity | Det. Smoke | Det. AOD |
| 28 | Det. Mask | Dew Point | LCC |
| 29 | Wind Direction | Friction Velocity | USWRF |
| 30 | U-wind | Det. Mask | Wind Direction |
| 31 | Visibility | DSWRF | % forest |
| 32 | LCC | LCC | Population |
| 33 | Wind Speed | Wind Speed | % developed |
| 34 | Gust | Rel. Humidity | Wind Speed |
| 35 | TCC | Gust | Gust |
